# Antimicrobial Resistance and Biofilm Formation of *Bordetella bronchiseptica* in Central China, with Evidence of a Rare Heteroresistance Strain to Gentamicin

**DOI:** 10.3390/ani14091301

**Published:** 2024-04-25

**Authors:** Li Yi, Haoran Fan, Shuo Yuan, Rishun Li, Haikun Wang, Yingying Quan, Hui Zhang, Yuxin Wang, Yang Wang

**Affiliations:** 1 College of Life Science, Luoyang Normal University, Luoyang 471934, China; lilili123168@163.com; 2 Henan Provincial Engineering Research Center for Detection and Prevention and Control of Emerging Infectious Diseases in Livestock and Poultry, Luoyang 471023, China; hr980402@163.com (H.F.); yuanshuo202206@163.com (S.Y.); ri_shun.li@haust.edu.cn (R.L.); 17630389653@163.com (H.W.); quan00826@163.com (Y.Q.); 3 College of Animal Science and Technology, Henan University of Science and Technology, Luoyang 471023, China; 4 China Animal Health and Epidemiology Center, Qingdao 266033, China; zhui0216@aliyun.com

**Keywords:** antimicrobial resistance, biofilm-forming ability, *B. bronchiseptica*, heteroresistance, pigs

## Abstract

**Simple Summary:**

*Bordetella bronchiseptica* (*B. bronchiseptica*) is a severe zoonotic pathogen widespread in aquaculture environments. However, information on the distribution and resistance of this pathogen remains scarce in pig farms. This study isolated and analyzed *B. bronchiseptica* from pig farms in central China and revealed a high frequency of resistance to this pathogen, with most strains forming biofilms. In addition, a *B. bronchiseptica* strain with a heteroresistant phenotype to gentamicin was isolated, which is a rare case and requires vigilance. This study enhances our understanding of the biological characteristics, such as the distribution and resistance, of *B. bronchiseptica* in pig farms in central China. This can provide valuable data for future related research and pathogen prevention and control.

**Abstract:**

*Bordetella bronchiseptica* is a significant contributor to respiratory disease in pigs, leading to substantial economic losses in the swine industry worldwide. We isolated 52 *B. bronchiseptica* strains from 542 samples collected from pigs with atrophic rhinitis and bronchopneumonia in central China. Multi-locus sequence typing identified two prevalent sequence types: ST6 (69.23%) and ST7 (30.77%). PCR-based detection of seven virulence genes (*fhaB*, *prn*, *cyaA*, *dnt*, *bteA*, *fla*, and *bfrZ*) revealed that six of these genes were present in over 90% of the isolates, with *bfrZ* being the exception at 59.62%. Antimicrobial susceptibility testing, performed using the K-B method, demonstrated high sensitivity to enrofloxacin, polymyxin, and doxycycline but a notable resistance to tylosin, trimethoprim, tobramycin, ciprofloxacin, and amikacin. Remarkably, 86.54% of the isolates exhibited a multidrug-resistant phenotype. Notably, we successfully screened a strain of *B. bronchiseptica* with a heteroresistance phenotype to gentamicin using population analysis profiling, which is a rare case. Biofilm-formation assays indicated that 96.15% of the isolates possessed biofilm-forming capabilities. These findings provide crucial insights into the prevalence of *B. bronchiseptica* in central China, facilitating the development of effective preventive measures to safeguard both animal and human health.

## 1. Introduction

*Bordetella bronchiseptica* (*B. bronchiseptica*), an aerobic Gram-negative coccobacillus, is a potential zoonotic pathogen that mainly causes respiratory diseases in humans and other animals [1]. Infected animals can transmit canine infectious respiratory syndrome, feline bronchitis, porcine atrophic rhinitis, and rabbit rhinitis [2,3]. In the pig industry, *B. bronchiseptica* is the primary pathogen causing porcine respiratory disease syndrome and porcine atrophic rhinitis. It causes mild clinical symptoms such as bronchitis, conjunctivitis, rhinitis, and mandibular lymphadenopathy. Severe symptoms include enlarged turbinates and severe pneumonia, which can lead to the delayed growth and development of pig herds, increasing elimination rates, thus causing serious economic losses to pig farms [4]. *B. bronchiseptica* is often co-infected with other pathogens, leading to herd diseases. For example, co-infection with *Pasteurella multocida* can result in atrophic rhinitis in herds [5]. Additionally, co-infection with *Streptococcus* and *Haemophilus parasuis* can enhance the respiratory colonization of the latter [6]. Moreover, it can interact with porcine reproductive and respiratory syndrome and swine influenza viruses, increasing the severity of respiratory pathological damage. *B. bronchiseptica* has become one of the most important pathogens seriously endangering the pig industry [7].

Antibiotics are widely used in treating bacterial diseases in humans and animals due to their high bactericidal ability. However, with the overuse of antibiotics, drug-resistant strains of bacteria, including *B. bronchiseptica*, may occur, rendering the associated diseases caused by them out of control [8]. We thus need to always have a clear understanding of them. Therefore, it is essential to conduct surveys on the distribution, antibiotic resistance, and virulence of pathogenic bacteria. Antibiotic resistance, especially multidrug resistance (MDR), is a public health concern [9]. MDR infections are associated with worse clinical outcomes and higher treatment costs compared to other infections. It is concerning that the emergence of widely antibiotic-resistant strains may render some infections untreatable [10,11]. According to relevant studies, antibiotic susceptibility testing reports a resistance phenotype known as heteroresistance, which falls between sensitivity and resistance [12]. Although the clinical relevance of heteroresistance is unclear, in today’s environment of antibiotic misuse, heteroresistant infections are more likely to evolve into antibiotic-resistant infections, leading to treatment failure [13].

This study aimed to enhance the understanding of biological characteristics such as antibiotic resistance in *B. bronchiseptica* and establish a foundation for future research on the evolution of antibiotic resistance phenotypes.

## 2. Materials and Methods

### 2.1. Bacterial Isolation and Identification

All of the clinical samples used in this study were submitted by veterinarians or farm owners to the Henan Engineering Research Center of Livestock and Poultry Emerging Disease Detection and Control, Henan University of Science and Technology (Luoyang, China) for routine testing. We collected 542 porcine bronchopneumonia and atrophic rhinitis samples from three different provinces (Henan, Hubei, and Hunan) in central China. The samples included nasal swabs (364) from sick pigs and lung tissue (178) collected from dead pigs during necropsy. About 10 g of each collected sample was taken and cut into pieces. Subsequently, the sample (0.2 g) was added to 1.8 mL of 0.9% saline and ground in a tissue homogenizer (TissueMaster™) until it became homogeneous. The tissue homogenate was used in a volume of 100 μL and applied on Tryptone Soya broth (TSB) agar plates. An aerobic culture was conducted at 37 °C for 24 to 36 h. Five colonies on the plate were selected with the same characteristics as *B. bronchiseptica* for identification [14]. Biochemical tests were performed according to previous methods for bacterial identification [15]. Presumptive isolates of *B. bronchiseptica* were finally confirmed using PCR amplification of the species-specific gene *fla* with the primers listed in Table 1 [16].

### 2.2. Multi-Locus Sequence Typing

Multi-locus sequence typing (MLST) was performed according to previous methods with some adaptations [16]. The isolates were analyzed using MLST, as described on the BIGSdb-Pasteur website (https://bigsdb.pasteur.fr/bordetella accessed on 5 March 2024). Bacterial DNA was extracted using a bacterial DNA extraction kit (TaKaRa MiniBEST Bacteria Genomic DNA Extraction Kit Ver.3.0). The amplification and sequencing of 7 house-keeping genes (*adk*, *fumC*, *glyA*, *tyrB*, *icd*, *pepA* and *pgm*) were performed following the method recommended by the website. The 7 allelic numbers were derived by comparing the 7 house-keeping genes of the isolates with the corresponding genes in the MLST typing website database. The sequence typings of the isolates were defined based on the 7 allelic numbers.

The 7 house-keeping gene sequences of *B. bronchiseptica* were concatenated and aligned in the database. Phylogenetic analysis was performed, and phylogenetic trees were constructed by means of the neighbor-joining method using Mega 11.0 with P-distance values and 1000 bootstrap replications.

### 2.3. Virulence Genes Detection

Seven well-characterized virulence factor-encoding genes, thought to be associated with host interactions, were screened in the isolates. These genes include *fhaB*, *prn*, *cyaA*, *dnt*, *bteA*, *fla*, and *bfrZ* [16,17]. The 7 pairs of virulence gene primers are listed in Table 1. Gene fragments were amplified using a PCR machine (Veriti™ 96-well PCR System, ThermoFisher, Thermo Fisher Scientific, Cambridge, MA, USA). The PCR conditions were as follows: preheating to 94 °C for 5 min, denaturation at 94 °C for 30 s, annealing at 59 °C for 30 s, elongation at 72 °C for 30 s and a final elongation step at 72 °C for 5 min. PCR products were analyzed via electrophoresis on a 1% agarose gel. The PCR products were sequenced to confirm the identities.

### 2.4. Antimicrobial Susceptibility Assay

A total of 13 types of antibiotics were prepared for antimicrobial susceptibility testing. The antibiotics tested in this study can be classified into eight categories: gentamicin (GEN), tobramycin (TOB), amikacin (AMK), enrofloxacin (ENR), ciprofloxacin (CIP), tilmicosin (TIL), tylosin (TYL), erythromycin (ERY), polymyxin B (PMB), doxycycline (DOX), trimethoprim (TMP), florfenicol (FFN), and amoxicillin (AMX). All determinations were performed using the K-B paper method recommended by the Clinical and Laboratory Standards Institute (CLSI) [18]. Appropriate concentrations of *B. bronchiseptica* were spread on an LB agar plate. This test used the reference strain *E. coli* ATCC 25,922 as a quality control organism. The diameter value of the inhibition ring was measured, and the presence of heteroresistance within the inhibition ring was also observed. Because specific clinic breakpoints for *B. bronchiseptica* are limited, we referred to the breakpoints for *Enterobacteriaceae* published in the CLSI document M100-ED32: 2022 for result interpretation in this study [16].

These *B. bronchiseptica* strains can be classified as multidrug-resistant (MDR) strains based on the criteria outlined by international experts in the provisional standard definition of acquired drug resistance [19].

### 2.5. Antimicrobial MIC Assay

According to the reference standard provided by the Clinical and Laboratory Standards Institute (CLSI) (2015 Edition), each antibiotic standard was prepared at concentrations ranging from 5120 to 10,240 µg/mL. Subsequently, each standard was dispensed into sterile EP tubes at 200 μL/tube. An appropriate volume of the dispensed standard stock solution diluted to the highest working concentration was tested during each experimental operation. Subsequently, the solution was diluted two-fold. Testing was performed according to the CLSI performance criteria for susceptibility testing of animal pathogens (VET01-S3). Following the measurement of the cultured bacterial fluid using McFarland turbidimetry, the bacterial fluid was diluted with saline to a concentration of 0.5 McFarland turbidity units. Then, the bacterial solution was diluted 1000-fold for later use. The final concentration achieved was 1 × 10^5^ CFU/mL. We added 100 μL of TSB broth to each well of a Type V 96-well plate. Following this, 100 μL of diluted stock standard solution was added to the wells starting from the first well of each row. The contents of the wells were mixed vigorously by blowing 5–10 times to ensure adequate mixing. We then pipetted 100 μL of the mixture into the second well, discarded 100 μL of the mixed solution after dilution in the 10th well and added 100 μL of spare bacterial solution into each well. Negative and positive controls were set up and repeated for each drug. Results were read by incubating in a 37 °C incubator for 16–20 h. Parallel testing was performed simultaneously using the control strain *E. coli* ATCC 25922. Susceptibility results for the test organisms were determined according to CLSI Performance Criteria for Antimicrobial Susceptibility Testing of Animal Pathogens (VET01-S3).

### 2.6. Antimicrobial Heteroresistance Assay

Population analysis profiling (PAP), a relatively quantitative colony counting method, was performed on suspected heteroresistant strains in the inhibition zone of the drug-sensitive paper diffusion method and is considered the gold standard for heteroresistance confirmation [20,21]. LB agar plates containing gentamicin 1.25–160 μg/mL in 8 concentration gradients, LB agar plates containing doxorubicin 0.3125–20 μg/mL in 8 concentration gradients and LB agar plates containing doxycycline 0.3125–20 μg/mL in 8 concentration gradients were prepared based on the MIC results obtained through the micro-broth dilution method. Single colonies of suspected heteroresistant strains were placed in LB liquid medium. The bacterial concentration was adjusted to 1 × 10^8^ CFU/mL after overnight incubation at 37 °C. Subsequently, 10-fold dilution was performed to obtain bacterial suspensions ranging from 10^7^ to 10^2^ CFU/mL. The appropriate bacterial solution was evenly coated on the PAP gradient antibiotic plate. Subsequently, bacterial inoculation of different concentrations of antibiotics on the plate and the blank control plate was completed sequentially. Three groups of replicates were performed for each plate. After incubation at 37 °C for 48 h, colonies were counted. The number of colonies was plotted against the relevant antibiotic reagent concentration using GraphPad Prism software. Identification was confirmed as heteroresistance if the ratio of the highest non-inhibitory concentration was more significant than 8 times the MIC value. The control strains were *B. bronchiseptica* ATCC 19395T and antibiotic-resistant *B. bronchiseptica* DY527.

### 2.7. Biofilm-Formation Ability Assay

The biofilm was quantified using the methods described in a previous study [22]. Isolates were incubated until they reached the logarithmic phase and were diluted to 1:100 in TSB. Eight wells of each *B. bronchiseptica* isolate from a 96-well plate were inoculated with 200 µL of bacterial suspension per well and then incubated at 37 °C in a constant temperature incubator. After 24 h, the bacterial solution was removed from the wells of the 96-well plate. The solution was aspirated, and 250 µL of deionized water was added to each well. The water was then aspirated to eliminate any remaining planktonic bacteria. Once the wells were air-dried, 250 µL of 100% formaldehyde fixative was introduced and aspirated after 15 min of fixation. Following natural air drying, 250 µL of 0.1% crystal violet staining solution was added to each well. The staining solution was aspirated after 10 min and 250 µL of deionized water was gently added to wash the wells. The water was then aspirated to remove any unbound crystal violet staining solution from the biofilm. After natural air-drying, 250 µL of 95% ethanol was added to dissolve any remaining staining. The crystal violet bound to the biofilm. After dissolution for 10 min, the absorbance value (OD_595 nm_) was measured repeatedly 3 times using a microplate reader and the mean value was calculated. The cut-off point (ODc) for biofilm formation was determined by detecting the negative control well’s optical density (OD) value twice using a spectrophotometer (Multiskan SkyHigh, ThermoFisher). Their ability to form biofilms was scored as follows: OD < ODc, absent producers; ODc < OD < 1.5 ODc, weak producers; 1.5 ODc < OD < 2 ODc, moderate producers; OD > 2 ODc, strong producers [23].

### 2.8. Statistical Analysis 

Statistical analysis was performed using the GraphPad Prism 8.0. All experiments were repeated three times and significant differences were analyzed by means of two-way analysis of variance (ANOVA). The experimental group was considered statistically significant if *p* < 0.05. 

## 3. Results

### 3.1. B. bronchiseptica Isolation and Identification

We isolated 52 *B. bronchiseptica* strains from 542 samples, resulting in an overall isolation rate of 9.59%. 

### 3.2. Multi-Locus Sequence Typing 

**Figure 1:**
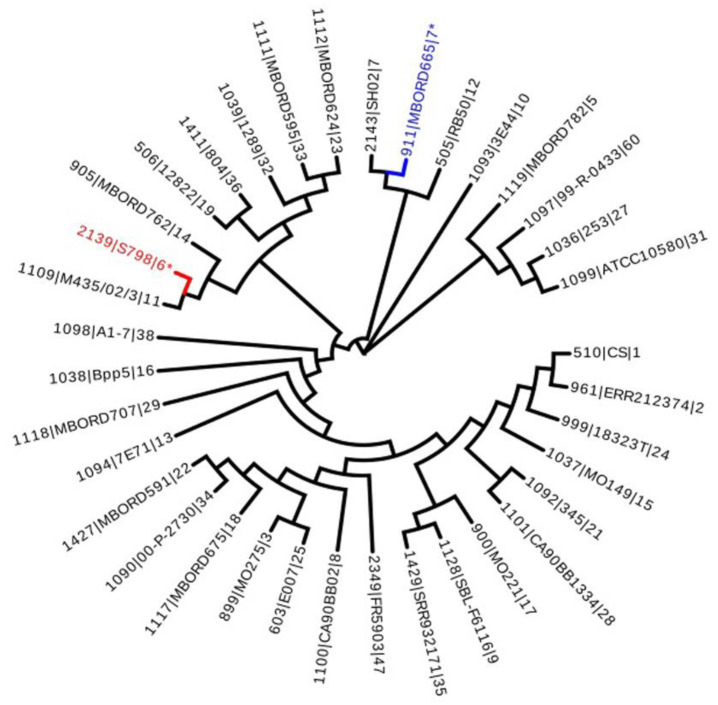
Phylogenetic tree based on multi-locus sequence typing in *B. bronchiseptica* isolates. * Indicate this experiment’s isolates. Reference strains are denoted by their respective accession numbers and their sequence typing.

MLST analysis showed that 52 isolates were classified into two sequence typings (STs), namely ST6 (69.23%, 36/52) and ST7 (30.77%, 16/52). The seven house-keeping gene sequences of each isolate of the two STs were concatenated for phylogenetic analysis to be compared in the database. The gene evolution tree is shown in Figure 1.

### 3.3. Virulence Factors Encoding Genes Detection

**Figure 2:**
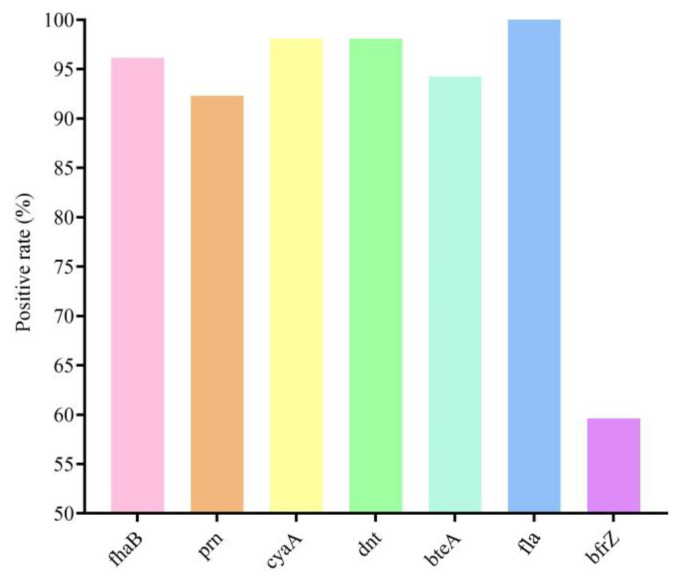
Positive detection rates of virulence genes in 52 *B. bronchiseptica* strains.

Screening of VFGs showed that the detection rates of *fhaB*, *prn*, *cyaA*, *dnt*, *bteA*, *fla* and *bfrZ* in this study were 96.15% (*n* = 50), 92.31% (*n* = 48), 98.08% (*n* = 51), 98.08% (*n* = 51), 94.23% (*n* = 49), 96.15% (*n* = 50), and 59.62% (*n* = 31), respectively (Figure 2). Of these isolates, 98.08% (*n* = 51) were positive for at least one of the seven VFGs detected, and of the VFG-positive isolates, 88.46% (*n* = 46) contained as many as six of the VFGs detected, and 51.92% (*n* = 27) contained all of the VFGs detected. 

### 3.4. Antimicrobial Susceptibility Testing

**Figure 3:**
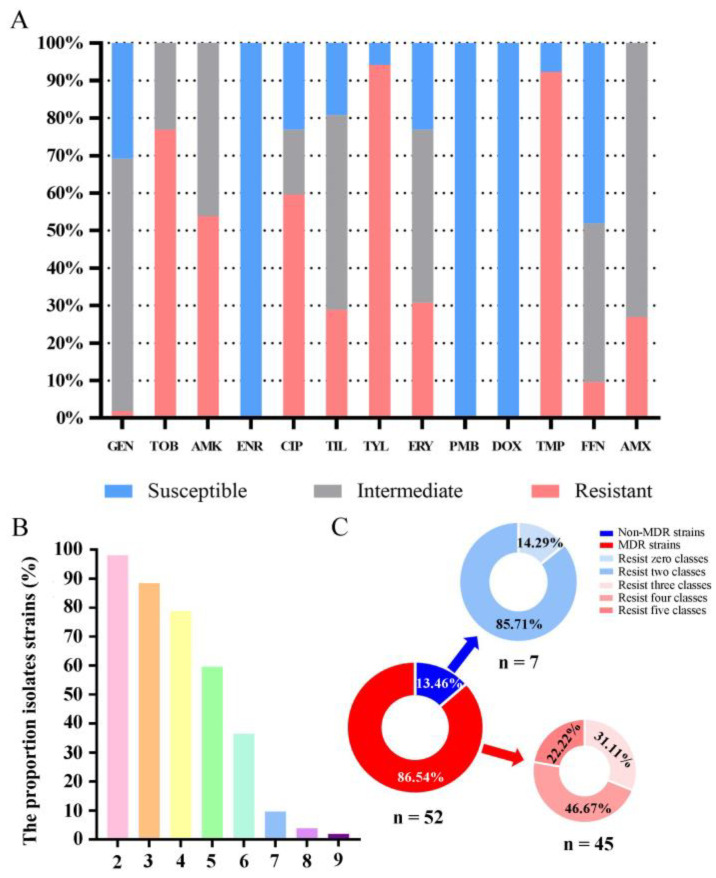
Antimicrobial susceptibility of *B. bronchiseptica* isolated from pigs in central China. (**A**) Percentage of isolates susceptible or resistant to the 13 kinds of antibiotics tested; (**B**) X axis shows the resistance of the isolates to at least several antibiotics, while the Y axis indicates the proportion of isolates; (**C**) percentages of MDR and non-MDR strains as well as percentage of strains resisting zero, two, three, four, and five classes of antibiotics.

The results show that isolates were resistant to TYL (94.23%, *n* = 49), TMP (92.31%, *n* = 48), TOB (76.92%, *n* = 40), CIP (59.62%, *n* = 52) and AMK (53.85%, *n* = 52) (Figure 3A). Among the 52 antibiotic-resistant isolates, the percentages of resistance to at least two, three, four, five, six, seven, eight and nine antibiotics were 98.08% (*n* = 51), 88.46% (*n* = 46), 78.85% (*n* = 41), 59.62% (*n* = 31), 36.54% (*n* = 19), 9.62% (*n* = 5), 3.84% (*n* = 2) and 1.92% (*n* = 1), respectively (Figure 3B). Moreover, all isolates were susceptible to GEN (100%, *n* = 52), PMB (100%, *n* = 52) and DOX (100%, *n* = 52). 

Among all the isolates, 13.46% (*n* = 7) were resistant to fewer than three classes of antibiotics, of which 14.29% (*n* = 1) were insensitive to zero classes of drugs, and 85.71% (*n* = 6) were insensitive to two classes of drugs. Most of the isolates in this study, 86.54% (*n* = 45), were not susceptible to more than three classes of antibiotics. These 45 strains of *B. bronchiseptica* can be classified as MDR strains. Among these MDR strains, the percentage of resistant strains to three, four, and five classes of drugs was 31.11% (*n* = 14), 46.67% (*n* = 21), and 22.22% (*n* = 10), respectively (Figure 3C). 

### 3.5. Antimicrobial MIC Testing

Comparing the various resistance phenotypes, it is evident that the overall MIC values remain relatively stable. Specifically, the overall MIC50 values of gentamicin, tilmicosin, trimethoprim, and florfenicol show a slight decreasing trend over time, while the overall MIC50 values of amikacin and erythromycin exhibit a slight increasing trend (Table 2).

### 3.6. Heteroresistance Test

The results of the K-B sensitivity test for gentamicin revealed that seven heteroresistant strains were identified initially, representing 13.46% of the total experimental strains. The K-B sensitivity test of polymyxin B revealed that eight heteroresistant strains were initially identified, representing 15.38% of the total strains. The results of the K-B sensitivity test for doxycycline revealed that four heteroresistant strains were initially identified, representing 7.69% of the total strains. The phenotype of heterogeneous resistance in strains is such that the dominant subpopulation exhibits “sensitivity” to antibiotics, while the subpopulation within the circle displays “resistance” to antibiotics.

**Figure 4:**
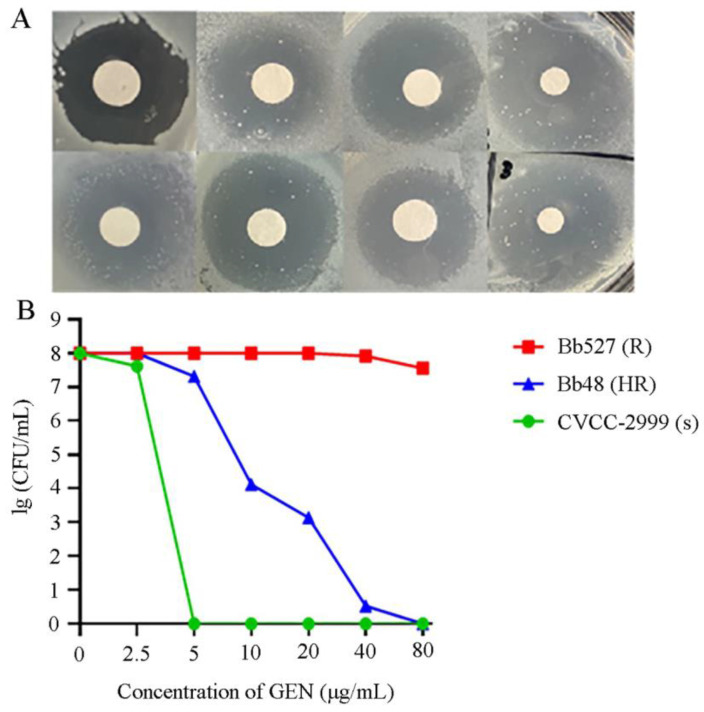
Heteroresistance test of *B. bronchiseptica*. (**A**) Preliminary screening for heteroresistant strains by disk diffusion. (**B**) The result of population analysis profile (PAP) for heteroresistant strains.

The PAP results verified the subclonal growth in the inhibition zone, indicating heteroresistance. It was established that one strain of *B. bronchiseptica* exhibited a heteroresistance phenotype to gentamicin, while the other strains identified in the initial screening were false positives. The colony profiling curves of these heteroresistant strains were between that of the *B. bronchiseptica* reference strain CVCC-2999 and that of the gentamicin-resistant strain Bb527 (Figure 4B). As the antibiotic concentration increases, the number of resistant subclones in the original strain that can grow on the plate gradually decreases, and none of the heteroresistant subpopulations have a minimum inhibitory concentration less than 8-fold higher than the original bacterial population. From the raw MIC values of this heteroresistant strain, it can be seen that it showed sensitivity to gentamicin. However, the PAP-AUC results revealed that the resistant subpopulations exhibited varying degrees of sensitivity to gentamicin, with a small number of subpopulations still growing on antibiotic plates with 8–16 times the MIC value. The MIC value of the colonies grown on the highest concentration of the gentamicin plate was compared with the gentamicin concentration of the plate (Table 2). The MIC value of the colonies on the plate with the highest gentamicin concentration for the strain (80 μg/mL) exceeded the gentamicin concentration of the plate (40 μg/mL). This suggests that the resistant subclones of the original heteroresistant strain could grow at 80 μg/mL or even higher concentrations of gentamicin, indicating increased resistance levels. 

### 3.7. Biofilm-Formation Ability Test

**Figure 5:**
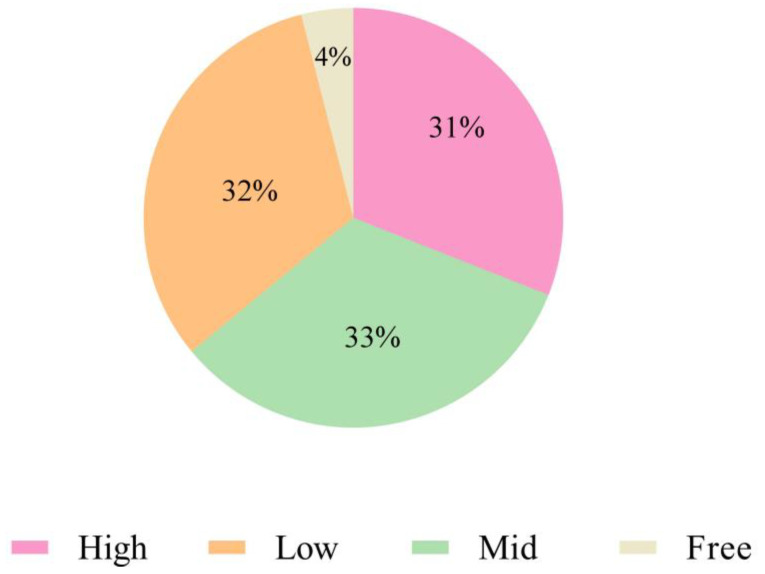
Detection of biofilm formation ability in 52 *B. bronchiseptica* strains.

In this study, 50 out of the 52 clinical isolates of *B. bronchiseptica* (96.15%) could form biofilms. Among them, 17 (32.69%) were firmly adherent, 16 (30.77%) were moderately adherent, 17 (32.69%) showed weak adherence, and 2 (3.85%) were non-adherent. Based on clinical characteristics, the isolates with a solid biofilm-forming ability were always highly pathogenic *B. bronchiseptica* (Figure 5).

## 4. Discussion

Although *B. bronchiseptica* is known to be a significant cause of respiratory diseases in pigs and an important causative agent of porcine atrophic rhinitis and several other animal respiratory diseases, there are few epidemiological reports on *B. bronchiseptica* worldwide, including China. We describe the isolation rate and various biological characteristics of *B. bronchiseptica* of swine origin in central China. Our samples came from central China between 2020 and 2022, including Henan, Hubei, and Hunan. Moreover, we isolated 52 clinical isolates of *B. bronchiseptica* from 542 samples, resulting in an average isolation rate of 9.59%. The MLST typing results indicated that the isolates belonged to ST6 and ST7. Zhang et al. isolated 89 strains of *B. bronchiseptica* from 2462 samples from the same provinces in China between 2018 and 2020, with an average isolation rate of 3.61% [8]. These results suggest that there may be an increasing risk of *B. bronchiseptica* outbreaks in China in the future. 

Essential virulence factors for *B. bronchiseptica* include *fhaB*, *prn*, *cyaA*, *dnt*, *bteA*, *fla*, and *bfrZ*. The expression of these virulence factors promotes the invasion of *B. bronchiseptica* in the host and enhances the bacterium’s virulence [16,17]. We tested the relevant genes encoding these seven VFGs, and surprisingly, the detection rate of six VFGs was higher than 90% (*fhaB*, 96.15%; *prn*, 92.31%; *cyaA*, 98.08%; *dnt*, 98.08%; *bteA*, 92.82%; *fla*, 96.15%), except for *bfrZ* (59.62%). Over 88% of the isolates contained six VFGs, while 51.92% contained seven VFGs. These results are also consistent with those reported in *B. bronchiseptica* and rabbit-derived *B. bronchiseptica* isolates of Chinese porcine origin [8,17], suggesting that the presence of these virulence factors exhibits a broad spectrum of characteristics. Based on the increasing isolation rate of *B. bronchiseptica* and the broad spectrum of virulence genes, we should invest more resources to study the pathogenic mechanisms of *B. bronchiseptica*. In addition, we need to develop corresponding prevention and control measures in advance to prevent it from threatening human health and pig industry development.

Antibiotics are one of the most effective tools for controlling *B. bronchiseptica* and other bacteria. However, the excessive use of antibiotics can result in the development of antibiotic-resistant bacteria, rendering the clinical use of antibiotics ineffective [24,25]. Therefore, continuous monitoring of resistance and trends in clinical microorganisms is crucial in many epidemiological studies [26,27,28]. In this study, we found that all isolates were susceptible to enrofloxacin (100%), polymyxin B (100%), and doxycycline (100%). These results are consistent with previous studies in other parts of China and other countries, such as Germany and Korea, suggesting that these antibiotics may be suitable candidates for treating *B. bronchiseptica* infections when necessary [8,16,24,29]. More isolates were resistant to tylosin (94.23%), trimethoprim (92.31%), tobramycin (76.92%), ciprofloxacin (59.62%), and amikacin (53.85%). These findings are also consistent with the results of other studies [8,16,30]. Therefore, these antibiotics are not recommended for use in clinical settings. Also of concern is the high prevalence of multidrug-resistant (MDR) isolates (86.54%). A *B. bronchiseptica* strain with a heteroresistant phenotype to gentamicin was identified in this study. This finding suggests that the routine administration of gentamicin at a concentration typically deemed effective in clinical treatment may need to be more efficacious. 

The biofilm plays a vital role in the colonization process of bacteria [31,32,33,34]. We analyzed the biofilm-forming ability of isolates from central China. Of the 52 isolates, 50 were able to form biofilms, indicating a high rate of biofilm formation among the isolates from central China. This may be due to the long-term use of low concentrations of antibiotics as growth promoters in the feed process of pigs [35,36]. Under the pressure of low antibiotic concentrations, *B. bronchiseptica* forming biofilm was selected, leading to a high rate of biofilm formation among isolates. This is likely the reason for the increased isolation and resistance rates of *B. bronchiseptica* of swine origin in central China. We also found that isolates with a solid ability to form biofilms tend to be highly pathogenic *B. bronchiseptica*. It is well-known that a biofilm is a self-protective substance that bacteria form to defend against external stress. This helps bacteria to effectively resist acid–base, temperature, and antibiotic challenges, thereby enhancing their survival rate in the host or environment [37]. Based on the biofilm’s protective mechanism, the bacteria’s ability to infect the host is greatly enhanced.

In summary, our results indicate that although most isolates of *B. bronchiseptica* of porcine origin in central China are still susceptible to gentamicin, polymyxin B, and doxycycline, more isolates are resistant to tylosin, trimethoprim, tobramycin, ciprofloxacin, and amikacin, with a high prevalence of MDR in the isolates. Therefore, to prevent the escalation of bacterial resistance and safeguard public food safety and health, it is imperative to regulate the use of antibiotics in food animals in the future to reduce the resistance of pathogens. The biofilm-forming ability of the present isolates exhibited a consistently high level, which could contribute to the elevated rates of isolation and resistance. In addition, we identified a strain with heteroresistance to gentamicin through PAP analysis. This is the first documented case of *B. bronchiseptica* being resistant to gentamicin. Therefore, this finding can offer valuable data on uncommon heteroresistance for future related studies.

## 5. Conclusions

In this study, we reported the isolation rate, virulence gene detection, antimicrobial resistance phenotype, VFGs and biofilm formation ability of *B. bronchiseptica* in pigs from central China. Our results show that *B. bronchiseptica* remains an important pathogen associated with respiratory disorders in pigs in China, and the isolation rate has increased at present. Over 88% of the isolates contained six VFGs, while 51.92% contained seven VFGs, indicating that the presence of these virulence factors exhibited a broad spectrum of characteristics. In terms of bacterial resistance, all isolates were found to be susceptible to ENR, PMB, and DOX in this study. Isolates showed more than 50% resistance to TYL, TMP, TOB, CIP, and AMX, while a *B. bronchiseptica* strain with a heterogeneous resistance phenotype to GEN was found. In addition, 50 of the isolates were able to form biofilms, indicating a high rate of biofilm formation among isolates. These isolates should receive more attention and further studies are necessary to monitor the prevalence of *B. bronchiseptica*.

## Figures and Tables

**Table 1:** Primers used in this study.

Primers	Primer Sequence (5′-3′)	Product Size (bp)
*fhaB* P1	GCGCAGAACATCACCAATG	475
*fhaB* P2	TGAAATACTCCATGGCGGAC	
*prn* P1	GACCTCGCTCAGTCGATC	555
*prn* P2	GAAGACATTCATGCGGAACAG	
*cyaA* P1	CTACGAGCAGTTCGAGTTTC	377
*cyaA* P2	TATTCATGTCGCCGTCGTA	
*dnt* P1	TGATCCTGCAGTGGTTGATC	491
*dnt* P2	ATCGGCATACGCCAGATC	
*bteA* P1	TGTTGAGCAACAACGTCAATC	474
*bteA* P2	TATGCAGGTCTTCGAGGTTC	
*fla* P1	AGGCTCCCAAGAGAGAAAGGCT	237
*fla* P2	TGGCGCCTGCCCTATC	
*bfrZ* P1	GCAATGACCTGAACCTGTATTT	368
*bfrZ* P2	CATGGGCATGTTCTTCTTGT	

**Table 2:** Summary of MIC values and frequency distributions for 13 antimicrobials tested with 52 *B. bronchiseptica* strains isolated from swine in central China.

Antibiotics	MIC Frequency Distribution (% of Isolates)					Antibiotics	MIC Frequency Distribution (% of Isolates)				
Gentamicin	≤4		8	≥16		Erythromycin	≤8		16	≥32	
	30.8%		67.3%	1.9%			23.1%		48.1%	28.8%	
Tobramycin	≤4		8	≥16		Polymyxin B	≤0.5	1	2	≥4	
	0		23.1%	76.9%			100%			0	
Amikacin	≤16		32	≥64		Doxycycline	≤2	4	8	≥16	
	0		46.2%	53.8%			100%		0	0	
Enrofloxacin	≤0.25		0.5	≥1		Trimethoprim	≤8			16	≥32
	100%		0	0			7.7%			92.3%	
Ciprofloxacin	≤0.125	0.25	0.5	1	≥2	Florfenicol	≤2		4	≥8	
	23.1%		17.3%	59.6%			48.1%		42.3%	9.6%	
Tilmicosin	≤5	10	20	≥40		Amoxicillin	≤8		16	≥32	
	19.2%		51.9%	28.9%			0		73.1%	26.9%	
Tylosin	≤24			48	≥96	Vertical red lines indicate the CLSI-approved breakpoint for susceptible, intermediate, and resistant in *B. bronchiseptica*; numbers in the lowest concentration of the tested antibacterial drug range represent the percentage of isolates that had MICs less than or equal to the lowest drug concentration tested per year, while numbers above the highest antibacterial drug concentration represent the percentage of isolates that had MICs greater than the highest drug concentration tested that year.					
	5.8%			94.2%							

## Data Availability

The data that support the findings of this study are available from the corresponding author upon reasonable request.
